# Multi-SNP Analysis of GWAS Data Identifies Pathways Associated with Nonalcoholic Fatty Liver Disease

**DOI:** 10.1371/journal.pone.0065982

**Published:** 2013-07-19

**Authors:** Qing-Rong Chen, Rosemary Braun, Ying Hu, Chunhua Yan, Elizabeth M. Brunt, Daoud Meerzaman, Arun J. Sanyal, Kenneth Buetow

**Affiliations:** 1 Center for Biomedical Informatics and Information Technology, National Cancer Institute, National Institutes of Health, Bethesda, Maryland, United States of America; 2 Biostatistics Division, Department of Preventive Medicine and Robert H. Lurie Comprehensive Cancer Center, Northwestern University, Evanston, Illinois, United States of America; 3 Department of Pathology & Immunology, School of Medicine, Washington University, St Louis, Missouri, United States of America; 4 Division of Gastroenterology, Hepatology, and Nutrition, Department of Internal Medicine, Virginia Commonwealth University Medical Center, Richmond, Virginia, United States of America; 5 Computational Science and Informatics Program, Complex Adaptive Systems Initiative, Arizona State University, Phoenix, Arizona, United States of America; Institute of Medical Research A Lanari-IDIM, University of Buenos Aires-National Council of Scientific and Technological Research (CONICET), Argentina

## Abstract

Non-alcoholic fatty liver disease (NAFLD) is a common liver disease; the histological spectrum of which ranges from steatosis to steatohepatitis. Nonalcoholic steatohepatitis (NASH) often leads to cirrhosis and development of hepatocellular carcinoma. To better understand pathogenesis of NAFLD, we performed the pathway of distinction analysis (PoDA) on a genome-wide association study dataset of 250 non-Hispanic white female adult patients with NAFLD, who were enrolled in the NASH Clinical Research Network (CRN) Database Study, to investigate whether biologic process variation measured through genomic variation of genes within these pathways was related to the development of steatohepatitis or cirrhosis. Pathways such as Recycling of eIF2:GDP, biosynthesis of steroids, Terpenoid biosynthesis and Cholesterol biosynthesis were found to be significantly associated with NASH. SNP variants in Terpenoid synthesis, Cholesterol biosynthesis and biosynthesis of steroids were associated with lobular inflammation and cytologic ballooning while those in Terpenoid synthesis were also associated with fibrosis and cirrhosis. These were also related to the NAFLD activity score (NAS) which is derived from the histological severity of steatosis, inflammation and ballooning degeneration. Eukaryotic protein translation and recycling of eIF2:GDP related SNP variants were associated with ballooning, steatohepatitis and cirrhosis. Il2 signaling events mediated by PI3K, Mitotic metaphase/anaphase transition, and Prostanoid ligand receptors were also significantly associated with cirrhosis. Taken together, the results provide evidence for additional ways, beyond the effects of single SNPs, by which genetic factors might contribute to the susceptibility to develop a particular phenotype of NAFLD and then progress to cirrhosis. Further studies are warranted to explain potential important genetic roles of these biological processes in NAFLD.

## Introduction

Nonalcoholic fatty liver disease (NAFLD) affects almost a third of the adult population in North America [1]. The clinical-histologic spectrum of NAFLD ranges from nonalcoholic fatty liver (NAFL) to nonalcoholic steatohepatitis (NASH) [2]. While NAFL progresses to cirrhosis in less than 5% of cases, NASH can progress to cirrhosis in 15–20% of cases [3]–[4]. NAFLD is also a risk factor for the development of hepatocellular cancer which can develop with or without cirrhosis [5]. It is therefore a public health priority to better understand the pathogenesis of the disease as well as factors that drive disease progression.

Recently, a single variant in *PNPLA* (rs738409; I148M) has been shown to be strongly associated with increased hepatic fat levels, inflammation and fibrosis [6]–[7]. Since the discovery of the association between the *PNPLA3* mutation and steatosis and steatohepatitis, several additional single nucleotide polymorphisms (SNPs) have been identified to be associated with NASH [7]. However, despite these individual SNP associations, the biologic mechanisms that distinguish alternative clinical outcomes or disease progression are largely unknown.

Genetic analysis of biologic processes as opposed to analysis of individual SNPs may provide more insight into pathogenesis. The pathway of distinction analysis (PoDA) is a recently developed computational technique which tests for the association of variation within multiple genes involved in a defined biologic pathway with a given phenotype [8]. This method may thus be used to investigate whether collections of constitutional genome variability within biologic processes determine the predisposition to develop steatohepatitis vs. steatosis or drive the progression to cirrhosis and hepatocellular cancer. Importantly, it identifies interactions between SNPs in driving a specific phenotype even when individually the SNPs may not be significantly related to the phenotype.

In this analysis, PoDA was performed on a genome wide association study dataset obtained from the NIDDK NASH Clinical Research Network (CRN) on 250 highly characterized adult female subjects with varying phenotypes of NAFLD [9]. The specific objectives of the study were to determine whether biologic process variation measured through genomic variation of genes within these networks was related to the development of steatohepatitis or cirrhosis. We further evaluated the relationships of variation within these biological pathways with the severity of the individual histologic parameters of NAFLD. The results demonstrate the potential relationship of genomic variability within key biologic pathways that correlate with both the individual histologic features of NAFLD, the presence of steatohepatitis and progression to cirrhosis.

## Materials and Methods

### The Population Studied

The genome wide association study (GWAS) was conducted on 250 highly characterized adult subjects with varying phenotypes of NAFLD, a subset of patients who were enrolled into the NAFLD Database Study of NASH CRN [9]. The database study was an observational cohort where no therapeutic interventions were undertaken. From this cohort, non-Hispanic, white, female adults were selected for the GWAS pilot study in order to reduce heterogeneity. The median age was 53 years (interquartile range: 46–60 years). The nature and clinical features of subjects in this cohort have also been published [2], [9].

Single SNP association with the phenotype of NAFLD from this GWAS has already been published [9]. This report represents an independent analysis of the GWAS dataset to identify genetic variations in biologic pathways associated with cirrhosis, NASH and the severity of the individual histologic features of NAFLD. The underlying assumption for the analysis was that inherited variations in genes in biologic pathways may determine the network functional status and thus the disease phenotype. The NAFLD Database study was approved by the IRB of each of the participating institutions of the NASH CRN. The GWAS was approved by the NASH CRN Steering Committee and was approved by the IRB at Cedars Sinai Medical Center, where the GWAS was performed. For each subject included in this analysis, detailed clinical information and data related to the liver histology was available. The histology was analyzed by the pathology committee of the NASH CRN and categorized using the NASH CRN scoring system as described previously [10].

### Genotyping

Genotyping was performed with the use of Illumina HumanCNV370-Quadv3 BeadChips as described previously [9] **.** Eight out of 250 samples were identified as outliers by principal component analysis (PCA) and were therefore removed with 242 samples remaining for the analysis. Additional filters applied to SNP data eliminated variants that did not show Hardy-Weinberg Equilibrium (P<1e-008) and minor allele frequency <0.02; resulting in a total of 324,623 SNPs for the analysis.

### Data Analysis

The Pathway of Distinction Analysis (PoDA) [8] was applied to the NAFLD genotype data for the following histologic phenotypes: steatohepatitis, NAFLD activity score (NAS) and its histologic components (steatosis, cytologic ballooning and lobular inflammation), fibrosis stage, and cirrhosis. Each of these phenotypes were analyzed as qualitative binary traits as described below: (1) Steatohepatitis: definite steatohepatitis (n = 56) vs. controls (steatohepatitis absent, n = 131). (2) NAS: high score (NAS ≥5, n = 114) vs. low scores (NAS <5, n = 124), Steatosis: moderate to severe (grades 2–3, n = 102) vs. mild to none (grades <2, n = 93), Lobular inflammation: moderate to severe (grades 2–3, n = 96) vs. mild to none (grades 0–1, n = 99), Ballooning: many ballooned cells (grade 2, n = 111) vs. controls with no ballooning (grade 0, n = 84). (3) Fibrosis stage (range 0–4): fibrosis (score ≥2, n = 130) and control (score <2, n = 108). (4) Cirrhosis: cirrhosis (n = 35) vs. control (n = 204). The total number of samples analyzed is less than the full sample set because of incomplete clinical data on the cohort that does not allow a full analysis, and “n” reflects the total number of subjects on whom the detailed clinical, histological and GWAS data were available.

To assess the potential impact of population stratification generating non-disease related associations, the population was examined for all SNPs included in the analysis. Stratification analysis was performed on each of 7 phenotypes using Principal Component Analysis, which was implemented using singular value decomposition algorithm of R. No evidence of stratification was found in any of these 7 phenotypes used in the study.

The PoDA analysis was run systematically to the pathways represented in NCI/Nature Pathway Interaction Database (PID) [11]. Associations between genes and SNPs were made using dbSNP build 129. A total of 95924 SNPs in the data could be associated with at least one of the pathways representing 4849 unique genes. The SNP showing the greatest magnitude of association with a given phenotype was selected to represent each gene for a given analysis. A total of 893 pathways were covered in the dataset with a minimum of 5 genes in each pathway. PoDA analysis tests for differences in variation between cases and controls by computing genetic distances based on the variation observed within and between each group. A distance score was computed in each pathway for each sample measuring that sample’s distance to the remaining cases relative to its distance to the remaining controls for the collection of gene-based SNPs that constitute a given pathway. The distinction score (DS) quantifying the differential distributions of distance scores between cases and controls were then computed for each pathway. Significance [p(DS)] was assessed by resampling “dummy” pathways of the same length and computing the fraction of greater DS scores as described previously [8]. Odds ratios [O.R.] were obtained by constructing a logistic regression model of case status as a function of S values which measures the sample’s relative distance from the remaining ones. P-values were then adjusted for the multiplicity of pathways using FDR adjustment [q(O.R.)].

## Results

The PoDA analysis was performed on the samples and SNPs that remained following the quality control processing of the original dataset from 250 subjects (see Materials and Methods for the details). The collections of SNPs in genes contained in Pathway Interaction Database (PID) [11] were examined for association with the following histological features of NAFLD that were recorded from Central Pathology Review: diagnosis of definite steatohepatitis, presence of cirrhosis, stage of fibrosis, grade of steatosis, ballooning and inflammation, and the NAFLD activity score (NAS) which is derived from the grade scores for ballooning, lobular inflammation, and steatosis. These histological parameters were either categorized to be absent or present; the severity of histological features that were scored was analyzed as a binary score as described in the Materials and methods section.

### Biologic Pathways Associated with Definite Steatohepatitis

**Figure 1:**
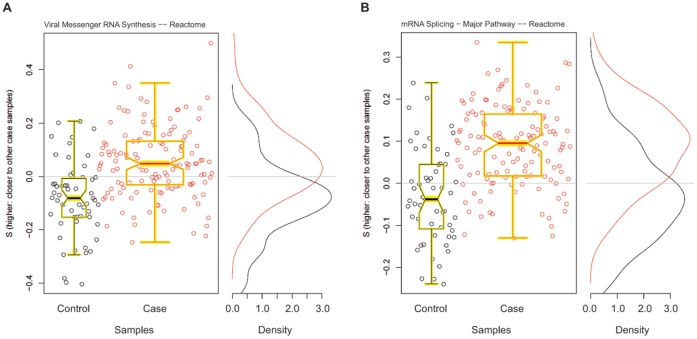
Two representative significant pathways in steatohepatitis. Scatter plots of distance score S for each pathway and overlayed with boxplots are given in the left panel; higher values of S indicate the sample is closer to other cases than it is to other controls. Distribution of S for cases (red) and controls (black) are given to the right. **A.** “Viral messenger RNA synthesis” – Reactome. **B.** “mRNA splicing – Major pathway” – Reactome.

Several pathways contained collections of SNPs that when examined simultaneously within the pathway were significantly associated with the histologic diagnosis of “definite steatohepatitis” (Table 1). The top two pathways were “Viral messenger RNA synthesis” and “Recycling of eIF2:GDP” (p(DS) values <0.001, and FDR adjusted odds ratio p-values of <0.01). Several biosynthesis pathways, such as “Terpenoid biosynthesis”, “Cholesterol biosynthesis”, “Pyrimidine biosynthesis”, “Biosynthesis of steroids”, “O-Glycan biosynthesis”, and “Bile acid biosynthesis”, were also significantly associated with the diagnosis of definite steatohepatitis. The SNP collection in Cell cycle and p53 signaling pathways were also found to be associated with the diagnosis of steatohepatitis. The scatter plots of distance scores for two representative pathways are provided in Figure 1. The gene-based variations in two pathways previously associated with HCC in a large Korean cohort [8], “Gamma-carboxylation, transport, and amino-terminal cleavage of proteins” and “Antigen processing and presentation”, were also associated with the diagnosis of definite steatohepatitis (p(DS) values = 0.034 and 0.037, respectively, with both showing FDR odds ratio adjusted p-values <0.01).

**Table 1:** Pathways significantly associated with NASH diagnosis.

Pathway	Source	No. of genesin pathway	No. of SNPsin pathway	p(DS)	O.R.	q(O.R.)	Significantin HCC
Viral Messenger RNA Synthesis	Reactome	65	46	0.000	>10	2.51E-06	
Recycling of eIF2:GDP	Reactome	8	5	0.000	2.58	6.11E-03	
mRNA Splicing - Major Pathway	Reactome	106	79	0.002	>10	2.63E-07	
Elongation of Intron-Containing Transcripts and co-transcriptional mRNA splicing	Reactome	44	37	0.002	>10	1.11E-05	
Cell cycle	KEGG	82	64	0.003	>10	8.51E-09	
Terpenoid biosynthesis	KEGG	6	6	0.003	4.73	4.22E-04	
p53 signaling pathway	BioCarta	13	12	0.004	>10	3.33E-05	
Leading Strand Synthesis	Reactome	9	6	0.004	4.50	2.05E-03	
inhibition of matrix metalloproteinases	BioCarta	8	7	0.006	7.30	2.65E-04	
Ethanol is oxidized by NAD+ to form acetaldehyde, NADH, and H+	Reactome	6	6	0.007	1.88	2.04E-03	
cell cycle: g2/m checkpoint	BioCarta	23	18	0.009	>10	1.42E-06	
regulation of transcriptional activity by pml	BioCarta	12	10	0.010	>10	1.25E-05	
Cholesterol biosynthesis	Reactome	8	8	0.012	>10	4.52E-05	
regulation of eif2	BioCarta	11	9	0.013	9.83	1.44E-04	
Telomere C-strand (Lagging Strand) Synthesis	Reactome	9	6	0.014	4.50	2.05E-03	
Folding of actin by CCT/TriC	Reactome	10	9	0.015	4.31	5.98E-03	
Toll Like Receptor 4 (TLR4) Cascade	Reactome	6	6	0.016	3.39	1.58E-03	
Lagging Strand Synthesis	Reactome	9	6	0.016	4.50	2.05E-03	
RNA Polymerase III Transcription Termination	Reactome	16	12	0.016	>10	1.53E-05	
Mitochondrial tRNA aminoacylation	Reactome	21	18	0.017	>10	1.16E-05	
Direct p53 effectors	NCI-Nature	139	117	0.022	>10	8.51E-09	
Pyrimidine biosynthesis (interconversion)	Reactome	11	7	0.022	3.50	1.29E-02	
Biosynthesis of steroids	KEGG	23	21	0.023	>10	8.69E-05	
DNA strand elongation	Reactome	9	6	0.023	4.50	2.05E-03	
Regulation of CDC42 activity	NCI-Nature	30	21	0.024	>10	1.18E-06	
mitochondrial fatty acid beta-oxidation of unsaturated fatty acids	Reactome	6	5	0.028	2.00	4.20E-03	
Processing of Capped Intron-Containing Pre-mRNA	Reactome	35	30	0.028	>10	4.06E-05	
Aurora B signaling	NCI-Nature	42	36	0.028	>10	8.69E-06	
eukaryotic protein translation	BioCarta	20	13	0.029	8.75	2.05E-03	
O-Glycan biosynthesis	KEGG	30	26	0.029	>10	6.89E-08	
the prc2 complex sets long-term gene silencing through modification of histone tails	BioCarta	14	11	0.031	>10	7.00E-05	
Bile acid biosynthesis	KEGG	31	29	0.032	>10	1.95E-05	
Regulation of retinoblastoma protein	NCI-Nature	65	57	0.033	>10	1.42E-07	
E2F transcription factor network	NCI-Nature	72	57	0.033	>10	4.11E-07	
Gamma-carboxylation, transport, and amino-terminal cleavage of proteins	Reactome	8	6	0.034	2.62	9.75E-03	yes
Trafficking of AMPA receptors	Reactome	5	5	0.034	1.14	3.41E-01	
Phosphorylation of CD3 and TCR zeta chains	Reactome	8	5	0.034	4.30	2.02E-04	
Antigen processing and presentation	KEGG	64	38	0.037	>10	1.22E-05	yes
Hemostasis	Reactome	5	5	0.040	>10	2.92E-08	
Prostate cancer	KEGG	80	67	0.040	3.10	1.28E-03	
deregulation of cdk5 in alzheimers disease	BioCarta	7	7	0.040	3.72	1.94E-03	
Processive synthesis on the C-strand of the telomere	Reactome	6	5	0.043	2.57	7.36E-03	
Electron Transport Chain	Reactome	66	48	0.043	>10	4.67E-06	
BARD1 signaling events	NCI-Nature	29	27	0.044	>10	1.59E-05	
cyclins and cell cycle regulation	BioCarta	23	20	0.045	>10	2.84E-05	
1- and 2-Methylnaphthalene degradation	KEGG	13	12	0.046	3.70	2.15E-03	
Aminoacyl-tRNA biosynthesis	KEGG	39	33	0.048	>10	6.02E-06	
trefoil factors initiate mucosal healing	BioCarta	37	31	0.048	>10	2.37E-06	
Nectin adhesion pathway	NCI-Nature	28	28	0.049	>10	8.58E-07	

Note: Pathway-length based resampled p-values, denoted as p(DS), are given for significant pathways (p<0.05), along with odds ratios and associated FDRs for a logistic regression model. The pathways previously shown to be associated with HCC are marked.

### Biologic Pathways Associated with Histologic Activity of NAFLD

We next investigated the relationship between pathway-based SNPs with NAS, and its independent components: steatosis, lobular inflammation and ballooning [10]. For this analysis, a NAS score ≥5 was used to identify high disease activity. A high NAS was associated with the SNP variants in “Glycoprotein hormones” (p(DS) value<0.001, FDR odds ratio adjusted p-value<0.01, Table S1). Interestingly there were multiple cancer pathways associated with NAS including “Colorectal cancer” (p(DS) = 0.002, FDR odds ratio adjusted p-value<0.01), “Renal cell carcinoma” (p(DS) = 0.011, FDR odds ratio adjusted p-value<0.01), “Pathways in cancer” (p(DS) = 0.021, FDR odds ratio adjusted p-value<0.01), “Melanoma” (p(DS) = 0.038, FDR odds ratio adjusted p-value<0.01), and “Acute myeloid leukemia” (p(DS) = 0.043, FDR odds ratio adjusted p-value<0.01). Of note, these pathways were not shown to be significantly associated with the diagnosis of definite steatohepatitis. Four pathways “Vibrio cholerae infection”, “Antigen processing and presentation”, “no2-dependent il-12 pathway in nk cells” and “ErbB signaling pathway” previously described to be associated with HCC were also associated with a high NAS [8].

The association of individual components of NAS with biologic pathways using a cutoff of p(DS) value<0.001 was nxt examined. “Glycoprotein hormones” and “il12 and stat4 dependent signaling pathway in th1 development” were observed to be associated with steatosis (Table S2). Of the previously described SNP variants in pathways associated with HCC, only “growth hormone signaling pathway” was associated with steatosis (p(DS) value = 0.002, FDR odds ratio adjusted p-value<0.01). “Hormone ligand-binding receptors” pathway was associated with lobular inflammation (Table S3) and “Terpenoid biosynthesis” was observed to be associated with ballooning (Table S4).

### Pathways Associated with Advanced Fibrosis and Cirrhosis

Analysis of fibrosis (stages 3–4 vs.0–2) showed that the top pathway that was associated with advanced fibrosis was the “p38 MAPK signaling pathway” (p(DS) value<0.001, FDR odds ratio adjusted p-value<0.01, Table S5). Two pathways “Galactose metabolism” and “Reelin signaling pathway” associated with HCC are also associated with fibrosis (p(DS) -values = 0.018 and 0.040 respectively, both with FDR odds ratio adjusted p-values <0.01).

**Figure 2:**
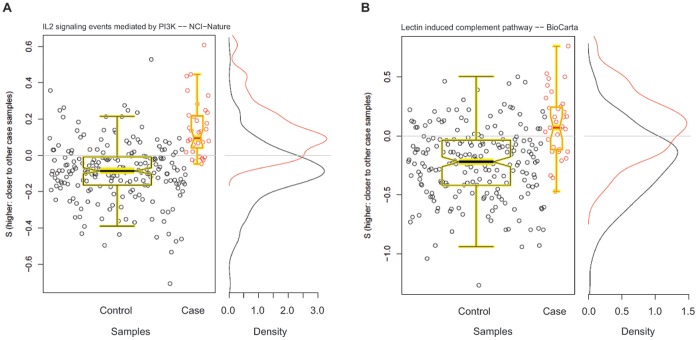
Two representative significant pathways in cirrhosis. Scatter plots of distance score S for each pathway and overlayed with boxplots are given in the left panel; higher values of S indicate the sample is close to other cases than it is to other controls. Distribution of S for cases (red) and controls (black) are given to the right. **A.** “IL2 signaling events mediated by PI3K” – NCI-Nature. **B.** “Lectin induced complement pathway” – BioCarta.

Extension of this analysis to cirrhosis versus absence of cirrhosis (Stage 4 fibrosis) identified several pathways that were associated with cirrhosis (Table 2). Three pathways were observed to be associated with cirrhosis with a p(DS) value<0.001, FDR odds ratio adjusted p-value<0.01: “Il2 signaling events mediated by PI3K”, “Mitotic metaphase/anaphase transition”, and “Protanoid ligand receptors”. The scatter plots of distance scores for two representative pathways are provided in Figure 2. Of note, these pathways were associated with the presence of cirrhosis but were not associated to fibrosis alone. The pathways “lectin induced complement pathway” and “signaling events mediated by stem cell factor (c-kit)” were also found to be associated with cirrhosis (p(DS) -values = 0.004 and 0.037, respectively, both with FDR odds ratio adjusted p-values <0.01) in previous HCC analysis [8].

**Table 2:** Pathways significantly associated with Cirrhosis.

Pathway	Source	No. of genes in pathway	No. of SNPs in pathway	p(DS)	O.R.	q(O.R.)	Significant in HCC
IL2 signaling events mediated by PI3K	NCI-Nature	37	31	0.000	>10	2.09E-07	
Mitotic Metaphase/Anaphase Transition	Reactome	8	5	0.000	>10	1.64E-05	
Prostanoid ligand receptors	Reactome	7	5	0.000	3.17	9.81E-03	
Recruitment of NuMA to mitotic centrosomes	Reactome	11	11	0.003	>10	8.70E-05	
COPI Mediated Transport	Reactome	10	10	0.003	>10	4.63E-04	
lectin induced complement pathway	BioCarta	12	12	0.004	>10	3.84E-06	yes
pertussis toxin-insensitive ccr5 signaling in macrophage	BioCarta	9	9	0.005	>10	1.20E-04	
granzyme a mediated apoptosis pathway	BioCarta	12	9	0.006	6.77	3.53E-04	
Synthesis of bile acids and bile salts via 27-hydroxycholesterol	Reactome	6	5	0.006	5.50	9.25E-05	
PDGFR-alpha signaling pathway	NCI-Nature	23	16	0.008	>10	3.75E-06	
RNA Polymerase I Promoter Opening	Reactome	17	12	0.008	6.21	2.14E-03	
Exocytosis of Alpha granule	Reactome	41	35	0.010	>10	3.73E-06	
Synthesis of bile acids and bile salts	Reactome	10	7	0.010	9.15	1.05E-04	
EPO signaling pathway	NCI-Nature	34	26	0.010	>10	2.30E-06	
Regulation of CDC42 activity	NCI-Nature	30	21	0.010	>10	7.12E-07	
E-cadherin signaling in the nascent adherens junction	NCI-Nature	35	33	0.013	>10	9.55E-07	
b cell survival pathway	BioCarta	13	11	0.015	>10	7.50E-05	
Arf6 trafficking events	NCI-Nature	40	35	0.017	>10	6.72E-07	
Recycling of eIF2:GDP	Reactome	8	5	0.018	2.07	3.25E-02	
role of mitochondria in apoptotic signaling	BioCarta	13	12	0.018	>10	1.43E-04	
Effects of Botulinum toxin	NCI-Nature	10	9	0.021	>10	2.04E-04	
IL2 signaling events mediated by STAT5	NCI-Nature	27	20	0.021	>10	8.10E-07	
IL2-mediated signaling events	NCI-Nature	55	48	0.023	>10	2.09E-07	
HDL-mediated lipid transport	Reactome	8	7	0.023	9.22	1.93E-04	
Caprolactam degradation	KEGG	7	6	0.024	5.43	1.02E-03	
eukaryotic protein translation	BioCarta	20	13	0.025	9.40	1.23E-03	
Glycosphingolipid biosynthesis - lacto and neolacto series	KEGG	26	22	0.028	>10	7.59E-06	
telomeres telomerase cellular aging and immortality	BioCarta	15	14	0.029	>10	5.92E-05	
Other semaphorin interactions	Reactome	14	12	0.031	>10	8.54E-06	
Beta oxidation of hexanoyl-CoA to butanoyl-CoA	Reactome	5	5	0.034	1.79	3.21E-02	
il22 soluble receptor signaling pathway	BioCarta	11	10	0.035	>10	9.10E-04	
C21-Steroid hormone metabolism	KEGG	10	9	0.035	5.02	6.60E-03	
Signaling events mediated by Stem cell factor receptor (c-Kit)	NCI-Nature	52	42	0.037	>10	1.31E-06	yes
Beta oxidation of octanoyl-CoA to hexanoyl-CoA	Reactome	5	5	0.039	1.81	1.57E-02	
Amino acid transport across the plasma membrane	Reactome	29	23	0.042	>10	3.73E-06	
p38 mapk signaling pathway	BioCarta	30	24	0.042	>10	8.18E-06	
Nef Mediated CD4 Down-regulation	Reactome	7	6	0.044	3.09	6.54E-03	
Fatty acid biosynthesis	KEGG	6	6	0.046	2.38	3.55E-03	
PDGFR-beta signaling pathway	NCI-Nature	54	45	0.046	>10	1.99E-07	
Internalization of ErbB1	NCI-Nature	34	31	0.047	>10	1.78E-06	

Note: Pathway-length based resampled p-values, denoted as p(DS), are given for significant pathways (p<0.05), along with odds ratios and associated FDRs for a logistic regression model. The pathways previously shown to be associated with HCC are marked.

### Pathways Associated with Multiple Features of NAFLD

NAFLD covers a wide clinical-histologic spectrum, ranging from steatosis to NASH of varying grades of activity and stages of fibrosis. To facilitate the identification of components that contribute to composite phenotypes and to identify common underlying pathways across different liver histologic manifestations of NAFLD, the pathways significantly associated with 2 or more histologic features (p(DS) value<0.05) are listed in Table 3 with their genes listed in Table S6. Not surprisingly, many pathways associated with steatosis, lobular inflammation or ballooning was also observed to be associated with NAS ≥5. With NAS ≥5 included, there are 4, 7 and 51 pathways associated with 4, 3 and 2 histological findings/scores respectively. The SNP variants in “Biosynthesis of steroids” and “Cholesterol biosynthesis” are associated with definite NASH, lobular inflammation, ballooning and the NAS; “Eukaryotic protein translation” was associated with definite NASH, ballooning, NAS and cirrhosis; “Terpenoid biosynthesis” was associated with definite NASH, lobular inflammation, ballooning and fibrosis. In addition, “Antigen processing and presentation” was associated with definite NASH and the NAS while “Recycling of eIF2:GDP” was associated with definite NASH, ballooning and cirrhosis.

**Table 3:** Pathways significantly associated with 2 or more phenotypes.

pathway (p(DS) <0.05)	Source	No. of genes in pathway	No. of SNPs in pathway	cirrhosis	fibrosis	nashdx	nas	steatosis	lobular	balloon	Significant in HCC	No. of phenotypes
Biosynthesis of steroids	KEGG	23	21	0	0	yes	yes	0	yes	yes	0	4
Cholesterol biosynthesis	Reactome	8	8	0	0	yes	yes	0	yes	yes	0	4
eukaryotic protein translation	BioCarta	20	13	yes	0	yes	yes	0	0	yes	0	4
Terpenoid biosynthesis	KEGG	6	6	0	yes	yes	0	0	yes	yes	0	4
Antigen processing and presentation	KEGG	64	38	0	0	yes	yes	0	0	0	yes	3
E2F transcription factor network	NCI-Nature	72	57	0	0	yes	yes	0	0	yes	0	3
Ethanol is oxidized by NAD+ to form acetaldehyde, NADH, and H+	Reactome	6	6	0	yes	yes	0	0	0	yes	0	3
Glycoprotein hormones	Reactome	8	7	0	0	0	yes	yes	yes	0	0	3
Hormone ligand-binding receptors	Reactome	11	10	0	0	0	yes	yes	yes	0	0	3
Pathogenic Escherichia coli infection	KEGG	32	27	0	0	0	yes	0	yes	yes	0	3
Recycling of eIF2:GDP	Reactome	8	5	yes	0	yes	0	0	0	yes	0	3
1- and 2-Methylnaphthalene degradation	KEGG	13	12			yes				yes		2
3-Chloroacrylic acid degradation	KEGG	13	13						yes	yes		2
angiotensin ii mediated activation of jnk pathway via pyk2 dependent signaling	BioCarta	34	28					yes	yes			2
B cell receptor signaling pathway	KEGG	58	47				yes		yes			2
BARD1 signaling events	NCI-Nature	29	27			yes			yes			2
Cell cycle	KEGG	82	64			yes				yes		2
cell cycle: g2/m checkpoint	BioCarta	23	18			yes				yes		2
Cellular roles of Anthrax toxin	NCI-Nature	22	17		yes					yes		2
DNA strand elongation	Reactome	9	6			yes				yes		2
Elongation of Intron-Containing Transcripts and co-transcriptional mRNA splicing	Reactome	44	37			yes				yes		2
ErbB signaling pathway	KEGG	88	75				yes				yes	2
Fatty acid biosynthesis	KEGG	6	6	yes						yes		2
Folding of actin by CCT/TriC	Reactome	10	9			yes				yes		2
Galactose metabolism	KEGG	26	22		yes						yes	2
Gamma-carboxylation, transport, and amino-terminal cleavage of proteins	Reactome	8	6			yes					yes	2
Gluconeogenesis	Reactome	15	11		yes					yes		2
growth hormone signaling pathway	BioCarta	22	18					yes			yes	2
inhibition of matrix metalloproteinases	BioCarta	8	7			yes				yes		2
intrinsic prothrombin activation pathway	BioCarta	23	19				yes	yes				2
lectin induced complement pathway	BioCarta	12	12	yes							yes	2
links between pyk2 and map kinases	BioCarta	26	22					yes	yes			2
mitochondrial fatty acid beta-oxidation of unsaturated fatty acids	Reactome	6	5			yes			yes			2
mRNA Processing	Reactome	9	7					yes	yes			2
mRNA Splicing - Major Pathway	Reactome	106	79			yes				yes		2
Nef Mediated CD4 Down-regulation	Reactome	7	6	yes						yes		2
Neuroactive ligand-receptor interaction	KEGG	27	23					yes	yes			2
Nicotinate metabolism	Reactome	12	11					yes	yes			2
no2-dependent il-12 pathway in nk cells	BioCarta	9	8				yes				yes	2
nuclear receptors coordinate the activities of chromatin remodeling complexes and coactivators to facilitate initiation of transcription in carcinoma cells	BioCarta	15	14		yes			yes				2
O-Glycan biosynthesis	KEGG	30	26			yes		yes				2
p53 signaling pathway	BioCarta	13	12			yes				yes		2
pertussis toxin-insensitive ccr5 signaling in macrophage	BioCarta	9	9	yes				yes				2
Processing of Capped Intron-Containing Pre-mRNA	Reactome	35	30			yes				yes		2
Reelin signaling pathway	NCI-Nature	29	25		yes						yes	2
Regulation of CDC42 activity	NCI-Nature	30	21	yes		yes						2
Regulation of gene expression in beta cells	Reactome	11	9						yes	yes		2
Regulation of Insulin Secretion by Glucagon-like Peptide-1	Reactome	10	9				yes		yes			2
Regulation of the Fanconi anemia pathway	Reactome	8	7				yes	yes				2
regulation of transcriptional activity by pml	BioCarta	12	10		yes	yes						2
regulators of bone mineralization	BioCarta	11	9				yes			yes		2
rho cell motility signaling pathway	BioCarta	32	28				yes		yes			2
role of nicotinic acetylcholine receptors in the regulation of apoptosis	BioCarta	17	14		yes					yes		2
Signaling events mediated by Stem cell factor receptor (c-Kit)	NCI-Nature	52	42	yes							yes	2
Synthesis of bile acids and bile salts	Reactome	10	7	yes						yes		2
Synthesis of bile acids and bile salts via 27-hydroxycholesterol	Reactome	6	5	yes						yes		2
Telomere C-strand (Lagging Strand) Synthesis	Reactome	9	6			yes				yes		2
Trafficking of AMPA receptors	Reactome	5	5			yes	yes					2
trefoil factors initiate mucosal healing	BioCarta	37	31			yes				yes		2
Vibrio cholerae infection	KEGG	47	39				yes				yes	2
Viral Messenger RNA Synthesis	Reactome	65	46			yes				yes		2
Vitamins	Reactome	6	5		yes				yes			2

Note: Pathways significantly associated with 2 or more histologic features (p(DS) value <0.05) are listed along with histologic feature, no. of genes and snps in pathway.

## Discussion

One approach to genetic analysis of complex diseases such as NAFLD is to stratify the characteristics of histology and identify the biologic mechanisms underpinning each. While recognizing that NAFLD represents an interaction between environmental, behavioral, and genetic factors, genetic analysis permits the identification of components that are modulated by the genetic makeup of the individual subject which is largely invariant over time. Recent GWAS and candidate-gene approaches are examples of this wherein a number of SNPs such as the *PNPLA3* have been identified that are associated with steatosis, steatohepatitis and more advanced disease [7], [12]. However, in the original publication from which the data is used for the current study has failed to identify any relationship between *PNPLA3* genetic variability and NAFLD, which may be a reflection of the modest sample size and highly select group of patients (non-Hispanic white female adults) [9]. In the current study, *PNPLA3* exists in the pathway “1- and 2-Methylnaphthalene degradation”, which is significantly associated with steatohepatitis and ballooning. Importantly, these studies have been able to de-link the relationship between insulin resistance and the hepatic histologic “phenotype” of NAFLD, indicating that genetic factors play an important role in determining the ultimate disease phenotype and outcomes of NAFLD.

The analysis performed here extends the genetic analysis concept by finding biologic pathways whose constitutional variations have the potential to stratify disease categories, underpin key subcomponents, and determine alternative clinical outcomes. Pragmatically, the PoDA method used here accomplishes this by incorporating the influence of SNPs that may not be significantly related to a disease phenotype individually but when present in combination with other such SNPs within a biologic network may be determinants of disease phenotype and outcomes [8]. Our study identifies several novel pathways, and implicitly gene-based SNPs, that appear to be associated with the presence and severity of several features of steatohepatitis. These data are primarily applicable to non-Hispanic white females because of the sample selection for this pilot GWAS study, therefore the generalizability of the conclusions remains an open question.

Some of the key pathways linked to the presence of steatohepatitis and disease progression include those related to cholesterol synthesis and protein translation. These may be particularly germane given the central role of the liver in cholesterol homeostasis and the fundamental importance of regulation of protein translation to maintain cell viability. NASH is associated with accumulation of free cholesterol without a corresponding increase in cholesterol esters [13]. This has recently been shown to be due to SREBP-2 driven transcriptional upregulation of HMG CoA reductase the rate-limiting enzyme for cholesterol synthesis [14]. The upstream components of cholesterol synthesis are also components of the mevalonate pathway the only part of the terpenoid synthesis pathway that exists in humans [15]. Several subcomponents of the terpenoid pathway and the cholesterol biosynthetic pathway e.g. farnesyl- and geranyl pyrophosphates affect cell proliferation and apoptosis [16]. The current studies provide additional evidence that genetic factors that may modulate the activity of these pathways may affect activation of cell injury and apoptotic pathways which drive the development of steatohepatitis.

Another cellular process that has been implicated in the development of NASH and its progression is the unfolded protein response (UPR) [17]. Inhibition of protein translation via phosphorylation of eIF-2α is a key step that relieves endoplasmic reticulum stress and increased eIF-2α phosphorylation has been seen in subjects with NASH [17]. Also, numerous microRNAs that are differentially activated in NASH target eiF-2α [18]. The identification of the protein translation pathway, of which eIF-2α is a key component, further corroborates the relevance of the protein translation machinery in the development of steatohepatitis and its progression to cirrhosis. It also indicates that susceptibility may be attributable to genetic variation within this pathway. Whether this occurs by altering miRNA expression and function, eIF structure and function or other mechanisms requires experimental elucidation.

It is also noteworthy that collections of SNPs in several cancer-related biological pathways were also identified to be related to disease activity. These pathways have several overlapping components including k-Ras, Wnt-β catenin and multiple kinases involved in pro-inflammatory and cell proliferative pathways [19]–[20]. These findings underscore the importance of the molecular pathways involved in cell proliferation and inflammation in defining the histologic activity of NAFLD and the susceptibility of these pathways to the genetic background of the individual. It is well known that cirrhosis is a risk factor for hepatocellular cancer and NASH-related cirrhosis is no exception to that rule [21]–[22]. Recently, hepatocellular cancer has been identified even in the absence of cirrhosis in subjects with NAFLD [5]. Our findings provide a rationale to further investigate the role of genetics in the development of HCC in such cases.

### Conclusion

NAFLD is a complex biological state with multiple histological phenotypes and varied progression to cirrhosis. While several genes have been identified to be associated with these phenotypes, this study identified additional biologic processes whose genetic variation may underpin alternative phenotypes and determine outcome. It identifies genetic variation in genes within pathways that while not significantly related to disease phenotype individually, in combination are related to the development of steatohepatitis (the aggressive form of NAFLD), disease activity as defined by liver histology and its progression to cirrhosis. It also identifies potential key cellular pathways that may define genetically susceptible individuals. Several of these pathways are already closely related to the pathogenesis of NASH and disease progression. Taken together, the results provide evidence for additional ways, beyond the effects of single SNPs, by which genetic factors might contribute to the susceptibility to develop a particular phenotype of NAFLD and then progress to cirrhosis. Further studies are warranted to explain potential important genetic roles of these biological processes in NAFLD.

## Supporting Information

Table S1 **Pathways significantly associated with NAS.**
(PDF)

Table S2 **Pathways significantly associated with Steatosis.**
(PDF)

Table S3 **Pathways significantly associated with Lobular inflammation.**
(PDF)

Table S4 **Pathways significantly associated with Ballooning.**
(PDF)

Table S5 **Pathways significantly associated with Fibrosis.**
(PDF)

Table S6 **List of SNP associated genes in pathways.**
(XLSX)
