# Supplementary material for: Multi-SNP Analysis of GWAS Data Identifies Pathways Associated with Nonalcoholic Fatty Liver Disease
Source: PLoS One. 2013 Jul 19;8(7):e65982. doi: 10.1371/journal.pone.0065982 (PMC3716806; doi:10.1371/journal.pone.0065982)
Supplement: Table S1 — Pathways significantly associated with NAS. (PDF) [file pone.0065982.s001.pdf]

Table S1. Pathways significantly associated with NAS

Pathway	Source	No. of genes in pathway	No. of SNPs in pathway	p(DS)	O.R.	q(O.R.)	Significant in HCC
Glycoprotein hormones	Reactome	8	7	0.000	2.56	1.22E-04	
Hormone ligand-binding receptors	Reactome	11	10	0.002	3.81	4.60E-05	
Apoptotic cleavage of cell adhesion proteins	Reactome	9	9	0.002	7.01	7.00E-05	
Colorectal cancer	KEGG	43	41	0.002	> 10	1.23E-09	
E2F transcription factor network	NCI-Nature	72	57	0.007	> 10	4.46E-10	
Cyclin D associated events in G1	Reactome	7	6	0.008	2.41	5.57E-03	
Epithelial cell signaling in Helicobacter pylori infection	KEGG	61	51	0.010	> 10	2.61E-09	
rho cell motility signaling pathway	BioCarta	32	28	0.010	> 10	8.61E-08	
Renal cell carcinoma	KEGG	60	55	0.011	> 10	5.98E-11	
regulators of bone mineralization	BioCarta	11	9	0.012	9.33	2.12E-06	
Transport of nucleosides and free purine and pyrimidine bases across the plasma membrane	Reactome	5	5	0.013	3.03	1.33E-03	
intrinsic prothrombin activation pathway	BioCarta	23	19	0.013	> 10	1.39E-06	
Regulation of the Fanconi anemia pathway	Reactome	8	7	0.015	2.85	6.99E-03	
Cholesterol biosynthesis	Reactome	8	8	0.016	4.64	1.20E-04	
Regulation of pyruvate dehydrogenase complex (PDC)	Reactome	6	5	0.019	2.10	3.11E-03	
Pathways in cancer	KEGG	268	241	0.021	> 10	1.22E-11	
Vibrio cholerae infection	KEGG	47	39	0.021	> 10	2.61E-09	yes
Antigen processing and presentation	KEGG	64	38	0.022	> 10	4.54E-07	yes
Natural killer cell mediated cytotoxicity	KEGG	125	93	0.023	> 10	1.69E-11	
btg family proteins and cell cycle regulation	BioCarta	9	8	0.026	4.15	7.11E-04	
B cell receptor signaling pathway	KEGG	58	47	0.027	> 10	1.68E-09	
role of mal in rho-mediated activation of srf	BioCarta	19	17	0.027	> 10	2.84E-04	
Regulation of Insulin Secretion by Glucagon-like Peptide-1	Reactome	10	9	0.028	4.77	3.38E-04	
Trafficking of AMPA receptors	Reactome	5	5	0.032	1.82	6.76E-03	
eukaryotic protein translation	BioCarta	20	13	0.036	4.43	2.95E-03	
Melanoma	KEGG	66	63	0.038	> 10	4.71E-10	
erythropoietin mediated neuroprotection through nf-kb	BioCarta	15	13	0.038	9.94	3.32E-05	
Taste transduction	KEGG	45	33	0.038	> 10	8.16E-05	
no2-dependent il-12 pathway in nk cells	BioCarta	9	8	0.041	3.98	3.89E-04	yes
signal transduction through il1r	BioCarta	37	29	0.041	> 10	1.95E-07	
ErbB4 signaling events	NCI-Nature	29	24	0.042	> 10	2.66E-07	
Acute myeloid leukemia	KEGG	56	49	0.043	> 10	2.59E-09	
Pathogenic Escherichia coli infection	KEGG	32	27	0.044	> 10	2.98E-04	
metabolism of anandamide an endogenous cannabinoid	BioCarta	7	6	0.046	2.87	6.62E-04	
ErbB signaling pathway	KEGG	88	75	0.047	> 10	1.69E-11	yes
Biosynthesis of steroids	KEGG	23	21	0.050	> 10	8.98E-05	
ras-independent pathway in nk cell-mediated cytotoxicity	BioCarta	21	16	0.050	6.31	4.85E-05	

Note: Pathway-length based resampled p-values, denoted as p(DS), are given for significant pathways ($p<0.05$), along with odds ratios and associated FDRs for a logistic regression model. The pathways previously shown to be associated with HCC are marked.
