# Supplementary material for: Multi-SNP Analysis of GWAS Data Identifies Pathways Associated with Nonalcoholic Fatty Liver Disease
Source: PLoS One. 2013 Jul 19;8(7):e65982. doi: 10.1371/journal.pone.0065982 (PMC3716806; doi:10.1371/journal.pone.0065982)
Supplement: Table S2 — Pathways significantly associated with Steatosis. (PDF) [file pone.0065982.s002.pdf]

Table S2. Pathways significantly associated with Steatosis

Pathway	Source	No. of genes in pathway	No. of SNPs in pathway	p(DS)	O.R.	q(O.R.)	Significant in HCC
Glycoprotein hormones	Reactome	8	7	0.000	> 10	1.45E-07	
il12 and stat4 dependent signaling pathway in th1 development	BioCarta	15	14	0.000	2.50	3.94E-05	
Hormone ligand-binding receptors	Reactome	11	10	0.002	5.11	7.77E-07	
growth hormone signaling pathway	BioCarta	22	18	0.002	> 10	4.32E-08	yes
estrogen responsive protein efp controls cell cycle and breast tumors growth	BioCarta	15	13	0.004	7.48	3.35E-05	
Generic Transcription Pathway	Reactome	16	14	0.005	9.59	2.62E-05	
il 6 signaling pathway	BioCarta	13	9	0.006	5.15	6.08E-05	
O-Glycan biosynthesis	KEGG	30	26	0.006	> 10	1.22E-09	
epo signaling pathway	BioCarta	11	8	0.006	3.86	4.96E-04	
trka receptor signaling pathway	BioCarta	13	10	0.007	8.37	4.16E-06	
FOXA1 transcription factor network	NCI-Nature	45	40	0.007	> 10	2.25E-10	
Neuroactive ligand-receptor interaction	KEGG	27	23	0.008	> 10	1.43E-07	
oxidative stress induced gene expression via nrf2	BioCarta	18	16	0.009	> 10	2.94E-06	
stat3 signaling pathway	BioCarta	8	6	0.009	3.76	2.98E-05	
Regulation of the Fanconi anemia pathway	Reactome	8	7	0.015	3.09	5.45E-03	
Alanine and aspartate metabolism	KEGG	28	26	0.017	> 10	9.99E-07	
Glyoxylate and dicarboxylate metabolism	KEGG	16	15	0.017	> 10	1.19E-05	
S1P2 pathway	NCI-Nature	26	25	0.017	> 10	4.09E-08	
the information processing pathway at the ifn beta enhancer	BioCarta	30	27	0.021	> 10	1.39E-06	
bioactive peptide induced signaling pathway	BioCarta	33	30	0.022	> 10	7.61E-08	
S1P3 pathway	NCI-Nature	28	28	0.022	> 10	1.35E-07	
RNA Polymerase I Transcription Initiation	Reactome	23	20	0.026	> 10	3.27E-05	
carm1 and regulation of the estrogen receptor	BioCarta	13	12	0.029	9.56	1.97E-05	
Signaling mediated by p38-alpha and p38-beta	NCI-Nature	35	31	0.029	> 10	3.83E-08	
ARMS-mediated activation	Reactome	6	6	0.029	2.65	7.98E-04	
regulation of eif-4e and p70s6 kinase	BioCarta	24	23	0.030	> 10	3.21E-07	
links between pyk2 and map kinases	BioCarta	26	22	0.032	> 10	1.39E-06	
chaperones modulate interferon signaling pathway	BioCarta	19	16	0.034	> 10	1.55E-05	
cbl mediated ligand-induced downregulation of egf receptors pathway	BioCarta	8	6	0.034	3.11	8.95E-05	
RNA Polymerase I Transcription Termination	Reactome	20	17	0.036	8.78	2.55E-04	
Dentatorubropallidoluysian atrophy (DRPLA)	KEGG	11	10	0.036	6.48	2.25E-05	
Sphingosine 1-phosphate (S1P) pathway	NCI-Nature	21	20	0.036	> 10	2.91E-07	
Nicotinate metabolism	Reactome	12	11	0.037	5.75	1.33E-04	
intrinsic prothrombin activation pathway	BioCarta	23	19	0.037	> 10	3.12E-06	
pertussis toxin-insensitive ccr5 signaling in macrophage	BioCarta	9	9	0.040	6.47	8.49E-05	
nuclear receptors coordinate the activities of chromatin remodeling complexes and coactivators to facilitate initiation of transcription in carcinoma cells	BioCarta	15	14	0.040	> 10	1.05E-05	
Frs2-mediated activation	Reactome	5	5	0.042	2.78	4.67E-04	
Sphingolipid metabolism	KEGG	40	32	0.043	> 10	1.47E-07	
NRIF signals cell death from the nucleus	Reactome	9	7	0.043	2.64	6.12E-03	
mRNA Processing	Reactome	9	7	0.046	2.06	1.76E-02	
angiotensin ii mediated activation of jnk pathway via pyk2 dependent signaling	BioCarta	34	28	0.046	> 10	2.42E-08	
Activation of DNA fragmentation factor	Reactome	6	6	0.049	2.87	2.17E-03	

Note: Pathway-length based resampled p-values, denoted as p(DS), are given for significant pathways ($p<0.05$), along with odds ratios and associated FDRs for a logistic regression model. The pathways previously shown to be associated with HCC are marked.
