# Supplementary material for: Multi-SNP Analysis of GWAS Data Identifies Pathways Associated with Nonalcoholic Fatty Liver Disease
Source: PLoS One. 2013 Jul 19;8(7):e65982. doi: 10.1371/journal.pone.0065982 (PMC3716806; doi:10.1371/journal.pone.0065982)
Supplement: Table S3 — Pathways significantly associated with Lobular inflammation. (PDF) [file pone.0065982.s003.pdf]

Table S3. Pathways significantly associated with Lobular inflammation

Pathway	Source	No. of genes in pathway	No. of SNPs in pathway	p(DS)	O.R.	q(O.R.)	Significant in HCC
Hormone ligand-binding receptors	Reactome	11	10	0.000	5.13	3.75E-06	
Drug metabolism - other enzymes	KEGG	53	39	0.003	> 10	2.50E-08	
Regulation of gene expression in beta cells	Reactome	11	9	0.004	9.17	8.07E-06	
Neuroactive ligand-receptor interaction	KEGG	27	23	0.004	> 10	6.70E-08	
beta-Alanine metabolism	KEGG	23	20	0.004	> 10	1.44E-07	
reversal of insulin resistance by leptin	BioCarta	10	8	0.004	6.44	1.51E-05	
Vitamins	Reactome	6	5	0.006	2.54	4.92E-04	
Prefoldin mediated transfer of substrate to CCT/TriC	Reactome	15	10	0.006	3.99	2.55E-03	
Cholesterol biosynthesis	Reactome	8	8	0.007	4.61	6.85E-05	
FOXA2 and FOXA3 transcription factor networks	NCI-Nature	44	41	0.007	> 10	2.22E-08	
Pyrimidine salvage reactions	Reactome	7	7	0.008	3.00	1.01E-03	
regulation of spermatogenesis by crem	BioCarta	10	10	0.010	7.72	9.18E-06	
mRNA Processing	Reactome	9	7	0.011	2.52	3.52E-03	
Ascorbate and aldarate metabolism	KEGG	26	19	0.011	> 10	6.17E-06	
pkc-catalyzed phosphorylation of inhibitory phosphoprotein of myosin phosphatase	BioCarta	21	20	0.012	> 10	1.15E-07	
Nicotinate metabolism	Reactome	12	11	0.013	8.55	1.89E-05	
Histidine metabolism	KEGG	31	27	0.013	> 10	7.26E-07	
Glycoprotein hormones	Reactome	8	7	0.014	2.43	1.21E-04	
Terpenoid biosynthesis	KEGG	6	6	0.015	3.42	4.66E-04	
Pathogenic Escherichia coli infection	KEGG	32	27	0.016	> 10	4.21E-05	
Cyclin A/B1 associated events during G2/M transition	Reactome	9	8	0.016	3.34	1.06E-03	
Propanoate metabolism	KEGG	33	29	0.018	> 10	3.32E-08	
Lysine degradation	KEGG	49	41	0.018	> 10	2.15E-07	
Cyclin E associated events during G1/S transition	Reactome	6	5	0.019	1.66	1.93E-02	
Calcineurin-regulated NFAT-dependent transcription in lymphocytes	NCI-Nature	49	43	0.019	> 10	3.59E-09	
rho cell motility signaling pathway	BioCarta	32	28	0.022	> 10	8.38E-08	
wnt lrp6 signalling	BioCarta	7	6	0.023	2.25	4.35E-03	
TRAIL signaling pathway	NCI-Nature	22	17	0.023	> 10	6.92E-05	
Golgi Associated Vesicle Biogenesis	Reactome	18	16	0.024	> 10	1.48E-05	
p38 signaling mediated by MAPKAP kinases	NCI-Nature	21	19	0.024	> 10	2.64E-06	
Cyclin A:Cdk2-associated events at S phase entry	Reactome	6	5	0.025	1.66	1.93E-02	
rac1 cell motility signaling pathway	BioCarta	36	32	0.026	> 10	1.64E-08	
Tryptophan metabolism	KEGG	48	41	0.028	> 10	5.13E-08	
3-Chloroacrylic acid degradation	KEGG	13	13	0.029	4.26	2.09E-04	
Striated Muscle Contraction	Reactome	7	7	0.029	3.38	8.13E-04	
Regulation of Insulin Secretion by Glucagon-like Peptide-1	Reactome	10	9	0.030	4.89	2.50E-04	
fas signaling pathway (cd95)	BioCarta	20	19	0.030	> 10	7.12E-07	
Lysosome Vesicle Biogenesis	Reactome	7	7	0.032	3.57	4.59E-04	
Signaling events regulated by Ret tyrosine kinase	NCI-Nature	39	34	0.033	> 10	2.15E-08	
Citrate cycle (TCA cycle)	KEGG	30	28	0.033	> 10	9.38E-07	
B cell receptor signaling pathway	KEGG	58	47	0.034	> 10	7.81E-09	
FAS signaling pathway (CD95)	NCI-Nature	35	33	0.034	> 10	1.31E-07	
Synthesis of DNA	Reactome	10	8	0.035	3.55	3.57E-03	
angiotensin ii mediated activation of jnk pathway via pyk2 dependent signaling	BioCarta	34	28	0.035	> 10	1.01E-07	
endocytotic role of ndk phosphins and dynamin	BioCarta	17	15	0.036	> 10	3.59E-05	
EphrinA-EPHA pathway	NCI-Nature	8	7	0.037	6.90	2.64E-06	
mitochondrial fatty acid beta-oxidation of unsaturated fatty acids	Reactome	6	5	0.039	1.74	2.96E-02	
Beta oxidation of decanoyl-CoA to octanoyl-CoA-CoA	Reactome	5	5	0.039	1.68	4.22E-02	
Intrinsic Pathway	Reactome	17	12	0.039	> 10	6.11E-06	
Biosynthesis of steroids	KEGG	23	21	0.040	> 10	6.46E-05	
links between pyk2 and map kinases	BioCarta	26	22	0.042	> 10	4.17E-07	
BARD1 signaling events	NCI-Nature	29	27	0.042	> 10	1.30E-05	
effects of calcineurin in keratinocyte differentiation	BioCarta	13	13	0.049	> 10	3.77E-06	
Further platelet releasate	Reactome	22	20	0.049	> 10	9.87E-05	

Note: Pathway-length based resampled p-values, denoted as p(DS), are given for significant pathways ($p<0.05$), along with odds ratios and associated FDRs for a logistic regression model. The pathways previously shown to be associated with HCC are marked.
