# Supplementary material for: Multi-SNP Analysis of GWAS Data Identifies Pathways Associated with Nonalcoholic Fatty Liver Disease
Source: PLoS One. 2013 Jul 19;8(7):e65982. doi: 10.1371/journal.pone.0065982 (PMC3716806; doi:10.1371/journal.pone.0065982)
Supplement: Table S4 — Pathways significantly associated with Ballooning. (PDF) [file pone.0065982.s004.pdf]

Table S4. Pathways significantly associated with Ballooning

Pathway	Source	No. of genes in pathway	No. of SNPs in pathway	p(DS)	O.R.	q(O.R.)	Significant in HCC
Terpenoid biosynthesis	KEGG	6	6	0.000	3.29	1.42E-03	
Viral Messenger RNA Synthesis	Reactome	65	46	0.001	> 10	5.28E-07	
trefoil factors initiate mucosal healing	BioCarta	37	31	0.002	> 10	1.26E-07	
eukaryotic protein translation	BioCarta	20	13	0.004	8.53	1.71E-04	
Fatty acid biosynthesis	KEGG	6	6	0.004	2.92	8.10E-04	
Cholesterol biosynthesis	Reactome	8	8	0.004	5.38	2.05E-04	
Drug metabolism - cytochrome P450	KEGG	72	54	0.005	> 10	1.64E-07	
p53 signaling pathway	BioCarta	13	12	0.007	> 10	8.05E-06	
Vitamin B6 metabolism	KEGG	5	5	0.007	2.52	1.63E-03	
Gluconeogenesis	Reactome	15	11	0.009	7.87	2.96E-04	
mRNA Splicing - Major Pathway	Reactome	106	79	0.009	> 10	6.02E-07	
Regulation of gene expression in early pancreatic precursor cells	Reactome	6	6	0.011	6.19	3.21E-05	
Recycling of eIF2:GDP	Reactome	8	5	0.012	2.96	5.00E-04	
Synthesis of bile acids and bile salts via 27-hydroxycholesterol	Reactome	6	5	0.013	2.66	2.06E-03	
E2F transcription factor network	NCI-Nature	72	57	0.013	> 10	5.49E-09	
Elongation of Intron-Containing Transcripts and co-transcriptional mRNA splicing	Reactome	44	37	0.013	> 10	2.27E-05	
Biosynthesis of steroids	KEGG	23	21	0.013	> 10	5.20E-05	
Regulation of gene expression in beta cells	Reactome	11	9	0.014	6.87	9.82E-05	
inhibition of matrix metalloproteinases	BioCarta	8	7	0.014	4.56	1.29E-04	
Ethanol is oxidized by NAD+ to form acetaldehyde, NADH, and H+	Reactome	6	6	0.016	1.79	3.43E-03	
melanocyte development and pigmentation pathway	BioCarta	14	12	0.016	> 10	3.33E-06	
role of nicotinic acetylcholine receptors in the regulation of apoptosis	BioCarta	17	14	0.019	> 10	1.61E-05	
regulators of bone mineralization	BioCarta	11	9	0.020	> 10	1.63E-06	
Cellular roles of Anthrax toxin	NCI-Nature	22	17	0.021	> 10	6.45E-06	
3-Chloroacrylic acid degradation	KEGG	13	13	0.022	4.05	1.06E-03	
Processing of Capped Intron-Containing Pre-mRNA	Reactome	35	30	0.023	> 10	3.23E-05	
Pathogenic Escherichia coli infection	KEGG	32	27	0.023	> 10	3.05E-04	
multiple antiapoptotic pathways from igf-1r signaling lead to bad phosphorylation	BioCarta	13	11	0.030	7.93	1.15E-04	
Glycine, serine and threonine metabolism	KEGG	44	35	0.031	> 10	5.88E-06	
Telomere C-strand (Lagging Strand) Synthesis	Reactome	9	6	0.031	2.03	6.40E-02	
adp-ribosylation factor	BioCarta	28	24	0.035	> 10	1.92E-06	
Integrin cell surface interactions	Reactome	52	44	0.035	> 10	5.58E-08	
SEMA3A-Plexin repulsion signaling by inhibiting Integrin adhesion	Reactome	8	7	0.036	2.82	3.18E-03	
fc epsilon receptor i signaling in mast cells	BioCarta	29	21	0.037	> 10	8.02E-06	
De novo synthesis of IMP	Reactome	6	6	0.037	2.59	3.01E-03	
Cell cycle	KEGG	82	64	0.037	> 10	9.89E-08	
cystic fibrosis transmembrane conductance regulator (cftr) and beta 2 adrenergic receptor (b2ar) pathway	BioCarta	20	18	0.037	> 10	4.80E-05	
Folding of actin by CCT/TriC	Reactome	10	9	0.037	3.60	7.10E-03	
Synthesis of bile acids and bile salts	Reactome	10	7	0.038	3.42	1.91E-03	
Nef Mediated CD4 Down-regulation	Reactome	7	6	0.042	3.09	1.24E-03	
Peptide ligand-binding receptors	Reactome	21	20	0.042	> 10	6.38E-05	
1- and 2-Methylnaphthalene degradation	KEGG	13	12	0.043	3.14	4.07E-03	
cell cycle: g2/m checkpoint	BioCarta	23	18	0.045	> 10	9.89E-06	
DNA strand elongation	Reactome	9	6	0.047	2.03	6.40E-02	

Note: Pathway-length based resampled p-values, denoted as p(DS), are given for significant pathways ($p<0.05$), along with odds ratios and associated FDRs for a logistic regression model. The pathways previously shown to be associated with HCC are marked.
