# Supplementary material for: Multi-SNP Analysis of GWAS Data Identifies Pathways Associated with Nonalcoholic Fatty Liver Disease
Source: PLoS One. 2013 Jul 19;8(7):e65982. doi: 10.1371/journal.pone.0065982 (PMC3716806; doi:10.1371/journal.pone.0065982)
Supplement: Table S5 — Pathways significantly associated with Fibrosis. (PDF) [file pone.0065982.s005.pdf]

Table S5. Pathways significantly associated with Fibrosis

Pathway	Source	No. of genes in pathway	No. of SNPs in pathway	p(DS)	O.R.	q(O.R.)	Significant in HCC
p38 MAPK signaling pathway	NCI-Nature	29	25	0.000	> 10	2.18E-08	
Olfactory transduction	KEGG	380	303	0.004	> 10	1.22E-09	
calcium signaling by hbx of hepatitis b virus	BioCarta	16	13	0.006	> 10	1.13E-05	
N-Glycan biosynthesis	KEGG	44	35	0.008	> 10	5.07E-07	
tumor suppressor arf inhibits ribosomal biogenesis	BioCarta	23	20	0.009	> 10	1.50E-06	
mapkinase signaling pathway	BioCarta	55	46	0.010	> 10	1.53E-09	
multi-drug resistance factors	BioCarta	6	5	0.012	1.63	6.91E-03	
Cysteine metabolism	KEGG	13	11	0.012	7.55	3.02E-05	
Retinol metabolism	KEGG	63	46	0.012	> 10	3.19E-07	
nfkb activation by nontypeable hemophilus influenzae	BioCarta	30	25	0.012	> 10	3.90E-08	
IL6-mediated signaling events	NCI-Nature	45	38	0.017	> 10	1.65E-08	
role of nicotinic acetylcholine receptors in the regulation of apoptosis	BioCarta	17	14	0.017	> 10	9.12E-06	
Galactose metabolism	KEGG	26	22	0.018	> 10	1.38E-06	yes
the 41bb-dependent immune response	BioCarta	14	13	0.019	9.87	2.82E-05	
Hypoxic and oxygen homeostasis regulation of HIF-1-alpha	NCI-Nature	18	17	0.019	> 10	4.65E-05	
nuclear receptors coordinate the activities of chromatin remodeling complexes and coactivators to facilitate initiation of transcription in carcinoma cells	BioCarta	15	14	0.021	> 10	1.14E-05	
Ethanol is oxidized by NAD+ to form acetaldehyde, NADH, and H+	Reactome	6	6	0.021	2.01	3.10E-04	
JNK signaling in the CD4+ TCR pathway	NCI-Nature	13	11	0.022	> 10	5.23E-06	
Terpenoid biosynthesis	KEGG	6	6	0.027	2.20	1.23E-02	
Insulin signaling pathway	KEGG	136	111	0.028	> 10	6.43E-11	
Vitamins	Reactome	6	5	0.028	2.61	3.70E-04	
mRNA Splicing - Minor Pathway	Reactome	40	31	0.036	> 10	4.51E-04	
Reelin signaling pathway	NCI-Nature	29	25	0.040	> 10	1.35E-07	yes
Global Genomic NER (GG-NER)	Reactome	20	17	0.043	7.29	4.30E-04	
Gluconeogenesis	Reactome	15	11	0.044	6.39	1.70E-04	
Cellular roles of Anthrax toxin	NCI-Nature	22	17	0.046	> 10	2.87E-05	
snRNP Assembly	Reactome	47	38	0.048	> 10	8.11E-05	
regulation of transcriptional activity by pml	BioCarta	12	10	0.049	5.96	1.43E-04	
IL1-mediated signaling events	NCI-Nature	36	30	0.049	> 10	2.41E-06	
cdk regulation of dna replication	BioCarta	18	13	0.049	5.34	5.22E-04	

Note: Pathway-length based resampled p-values, denoted as p(DS), are given for significant pathways ($p<0.05$), along with odds ratios and associated FDRs for a logistic regression model. The pathways previously shown to be associated with HCC are marked.
